# Antibiotic prescription preferences in paediatric outpatient setting in Estonia and Sweden

**DOI:** 10.1186/2193-1801-2-124

**Published:** 2013-03-21

**Authors:** Jana Lass, Viveca Odlind, Alar Irs, Irja Lutsar

**Affiliations:** Institute of Microbiology, Tartu University, Tartu, Estonia; Pharmacy Department, Tartu University Clinics, Tartu, Estonia; Medical Products Agency, Uppsala, Sweden; Division of Clinical Pharmacology, Tartu University, Tartu, Estonia; State Agency of Medicines, Tartu, Estonia

## Abstract

Aims of the study were to compare the paediatric outpatient antibiotic use in two countries with low overall antibiotic consumption and antibacterial resistance levels - Sweden and Estonia - and to describe the adherence to Estonian treatment guideline.

All prescriptions for systemic antibiotics for children less than 18 years during 2007 from the Swedish Prescribed Drug Register and Estonian Health Insurance Fund database were identified to conduct a descriptive drug utilisation study.

The total paediatric antibiotic use was 616 and 353 per 1000 in Estonia and Sweden, respectively. The greatest between country differences occurred in the age group 2 to 6 years –Estonian children received 1184 and Swedish children 528 prescriptions per 1000. Extended spectrum penicillin amoxicillin (189 per 1000) or its combination with beta-lactamase inhibitor (81 per 1000) and a newer macrolide clarithromycin (127 per 1000) were prescribed most often in Estonia whereas narrow spectrum penicillin phenoxymethylpenicillin (169 per 1000) and older generation macrolide erythromycin (21 per 1000) predominated in Sweden. For acute bronchitis, 17 different antibiotics (most commonly clarithromycin) were prescribed in Estonia despite the guideline recommendation not to use antibiotics.

The higher rate of antibiotic use especially of extended spectrum antibiotics in Estonia compared to Sweden emphasizes the need for national activities to promote appropriate use of antibiotics while treating children, even when the overall antibiotic consumption is low.

**Electronic supplementary material:**

The online version of this article (doi:10.1186/2193-1801-2-124) contains supplementary material, which is available to authorized users.

## Introduction

Antibiotics are among the most prescribed medicines in children across Europe including Estonia and Sweden (Olsson et al. 2011; Lass et al. 2011; Clavenna & Bonati 2009) but great quantitative and qualitative variations in their prescription profile have been shown between countries (Clavenna & Bonati 2011; Rossignoli et al. 2007).

As of many examples for quantitative differences, in Italy the antibiotic prescription rate was twice as high compared with Denmark (Lusini et al. 2009). Also the children in British Columbia received substantially more antibiotic prescriptions than Danish counterparts (Marra et al. 2007) and in the Netherlands the prevalence of the use of anti-infective drugs in children was much lower than in the UK and Italy (Sturkenboom et al. 2008). At the same time, studies for qualitative variations of prescribing antibiotics to children are outnumbered. Only limited data of few countries are available on the most frequently prescribed antibiotics (Clavenna & Bonati 2011; Rossignoli et al. 2007).

Amoxicillin has been reported to be the most frequently prescribed antibiotic in the Netherlands and Canada whereas the use of amoxicillin with clavulanic acid prevailed in Italy (Clavenna & Bonati 2011).

Estonia and Sweden are Northern European countries with relatively low antibiotic resistance among microorganisms commonly managed in outpatient setting (European Centre for Disease Prevention and Control 2009). Also the total antibiotic use in outpatient settings in Estonia and Sweden has been shown to be similar. In fact, when expressed in defined daily doses per 1000 inhabitants in 2002, total antibiotic use was lower in Estonia than in Sweden (Goossens et al. 2005).

Almost two decades ago, a study comparing antibiotic use in Swedish and Estonian university hospitals found that the frequency of antibiotic use in these countries was in general similar but differences in the prescription preferences were observed (Kiivet et al. 1998).

We are not aware of studies specifically comparing paediatric antibiotic prescription preferences in these countries.

Most antibacterial drugs are prescribed to children for the treatment of common paediatric conditions such as upper respiratory tract infections and acute otitis media (AOM) (Lusini et al. 2009; Finkelstein et al. 2000). Both conditions are often self-limiting viral infections, typically not benefitting from antibiotic therapy (Rossignoli et al. 2007; Moro et al. 2009; Hare et al. 2006) and are thus major causes of inappropriate antibiotic prescribing in the outpatient setting (Nitzan et al. 2010) and potential targets for interventions to improve the use of antibiotic medicines. Sweden has a nationwide government-funded multidisciplinary programme STRAMA (http://www.strama.se) to fight against the over- and misuse of antibiotics, no similar programme exists in Estonia. As a positive result, after initiation of STRAMA, the outpatient paediatric antibiotics use fell 34% in Sweden between the years 1992 and 2002 from 1159 to 764 prescriptions per 1000 (Högberg et al. 2005).

The first aim of this report was to compare the general antibiotic consumption rates and prescription preferences in paediatric outpatient settings in Estonia and Sweden. Secondly, we describe the selection of antibiotics for the most common diagnoses by physicians in Estonia and adherence to treatment guideline.

## Methods

### Setting

Estonia and Sweden both have government supported public health services. In Estonia, the general practitioners (GP) provide primary care and access to health care is fully covered to all subjects below 19 years. Antibiotics are fully reimbursed for children younger than 4 and 90% of the price of medicines is reimbursed from 4 to 16 years. Exceptionally, for cystic fibrosis patients, 100% of the ciprofloxacin price is reimbursed regardless of age.

In Sweden, the cost of antibiotics – like most other prescription medicines – is partly reimbursed. If the total cost for medicines during one year has surpassed a ceiling of SEK 1800 (around €200), the cost is fully reimbursed.

In both countries antibiotics are exclusively prescription medicines.

### Design

We conducted a descriptive drug utilisation study based on the Estonian Health Insurance Fund (EHIF) and Swedish Prescribed Drug Register (SPDR) database. Both are nationwide prescription databases, containing electronically submitted data of all prescription medicines dispensed by the pharmacies to individuals receiving ambulatory care. The Estonian database contains individual patient and physician identification numbers and is diagnosis-linked. The Swedish database contains product identification and patient’s age but no information with regard to dose or indication.

We identified all prescriptions for systemic antibacterial drugs (Anatomical Therapeutic Chemical (ATC) code J01) released for children aged less than 18 years between January 1 and December 31, 2007, from both databases.

We used the following data from the EHIF: patient’s age, prescriber speciality, drug data (package code, ATC Code, name of the active substance, brand name) and information on subsequent diagnoses.

From the Swedish database aggregated data on the number of prescriptions for each active substance for each age group was obtained.

We stratified the children by age based on the International Conference of Harmonization guideline as newborns and infants (0-1,99 years), pre-school children (2–5,99 years), school children (6-11,99 years) and adolescents (12–17,99 years) (2000).

Population data were obtained from the Statistics Estonia (http://pub.stat.ee/px-web.2001/Database/Rahvastik/databasetree.asp) and from Statistics Sweden (http://www.scb.se).

We used the Estonian national guideline for diagnosing and treating common infections in outpatient settings in Estonia, approved in 2005 http://www.ravijuhend.ee.

In Sweden, recent national guidelines on the treatment of acute otitis media, rhinosinusitis, pharyngotonsillitis and lower respiratory infections have been published by the Medical Products Agency (http://www.lakemedelsverket.se).

### Data analysis

We expressed the paediatric antibiotic use as number of prescriptions for systemic antibiotics (ATC code J01) per 1000 children aged until 18 years (included) in the population/year and calculated the 95% confidence intervals (CIs) by using R64 software (http://www.r-project.org/).

## Results

### Antibiotic consumption - Estonia versus Sweden

**Figure 1:**
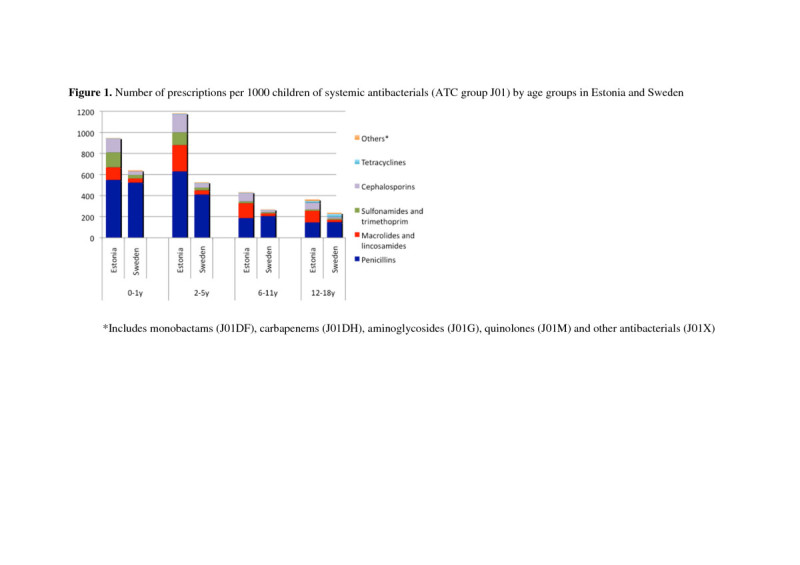
**Number of prescriptions per 1000 children of systemic antibacterials (ATC group J01) by age groups in Estonia and Sweden.** *Includes monobactams (J01DF), carbapenems (J01DH), aminoglycosides (J01G), quinolones (J01M) and other antibacterials (J01X).

The paediatric population in 2007 was 7.5 times smaller in Estonia (n = 258,515) as compared with Sweden (n = 1, 933, 920). At the same time the total paediatric antibiotic use was almost twice as high - 616 per 1000 (95% CI 613 to 619) in Estonia versus 353 per 1000 (95% CI 352 to 354) in Sweden (Figure 1).

The highest prescription rate in Estonia was found among 2 to 6 year old children whereas in Sweden it was highest among those less than 2 (Figure 1). The greatest difference between the two countries occurred in preschool children (age 2 to 6y) – the Estonian children received more than twice as many prescriptions compared with their Swedish counterparts (1184 vs. 528 per 1000 children). Adolescents had the lowest rate of antibacterial prescriptions in both countries but the difference in favour of Sweden as in all other age groups was observed.

### Prescription by antibiotic classes

A total of 55 different active substances (22 in Estonia and 50 in Sweden) were used. However, 90% of prescriptions were covered by 8 agents in both countries.

**Figure 2:**
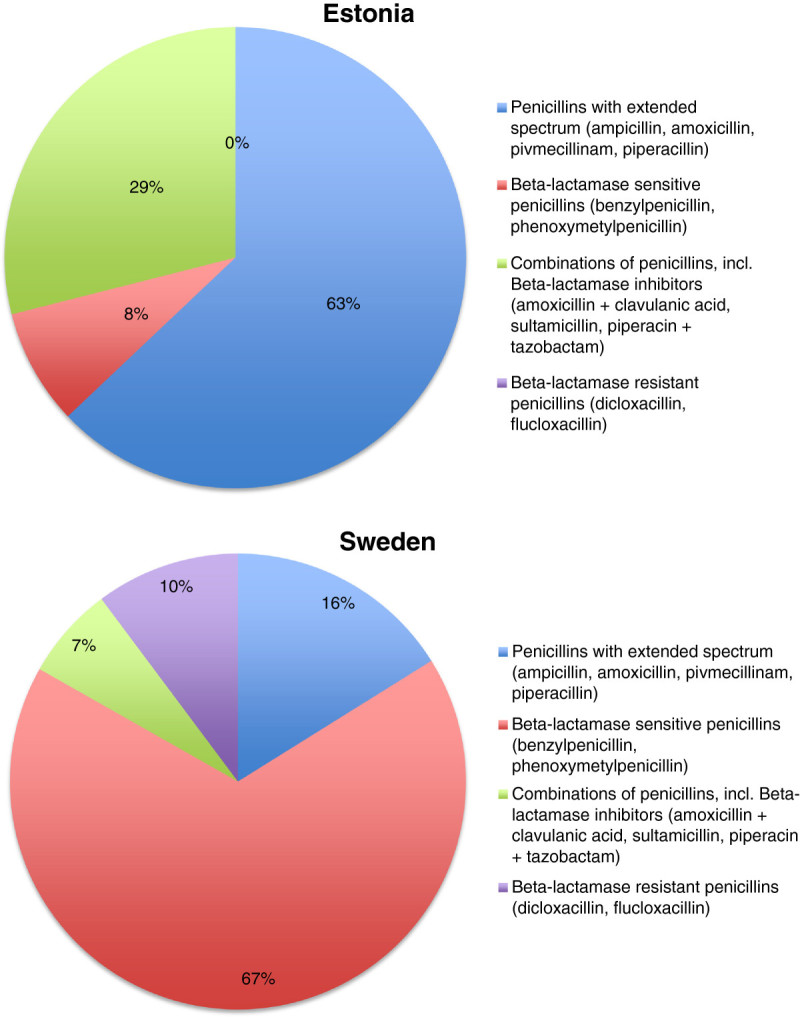
**Proportion of different penicillins used in Estonia and Sweden.**

Penicillins were the most widely prescribed antibiotics with the similar prescription rate in both countries (Figure 1) but the ratio of penicillins of all prescriptions was significantly greater in Sweden than in Estonia (74% vs. 49%). In addition, the qualitative selection of penicillins differed considerably - extended spectrum penicillin amoxicillin or its combination with beta-lactamase inhibitor (amoxicillin + clavulanic acid) were commonly prescribed in Estonia whereas narrow spectrum penicillins (e.g. phenoxymethylpenicillin) predominated in Sweden (Figure 2, Table 1). Penicillinase resistant penicillins (dicloxacillin and flucloxacillin) had a considerable use in Sweden, but were not available in Estonia.

**Table 1:** **Antibiotic agents prescribed most commonly - number of prescriptions per 1000 children and percentage of all prescriptions (in the brackets)**

	Estonia		Sweden	
1	Amoxicillin	189 (30.6)	Phenoxymethylpenicillin	169 (49.7)
2	Clarithromycin	127 (20.6)	Amoxicillin	35 (10.3)
3	Amoxicillin + clavulanic acid	81 (13.2)	Flucloxacillin	26 (7.5)
4	Sulfamethoxazol + trimethoprim	46 (7.4)	Cefadroxil	23 (6.6)
5	Cefprozil	35 (5.6)	Erythromycin	21 (6)
6	Cefuroxime	34 (5.5)	Amoxicillin + clavulanic acid	16 (4.9)
7	Cefadroxil	32 (5.2)	Trimethoprim	9 (2.7)
8	Phenoxymethylpenicillin	24 (4)	Sulfamethoxazol + trimethoprim	7 (2)
9	Azitromycin	15 (2.4)	Pivmecillinam	6 (1.6)
10	Doxycyclin	6 (1)	Doxycyclin	5 (1.6)
	Others	28 (4.5)	Others	24.9 (7)

Macrolides accounting for 24% of prescriptions were extensively used in Estonia (149 prescriptions per 1000) in all age groups, with the highest rates observed among children aged 6 to 17 years whereas in Sweden they were used less frequently (29 prescriptions per 1000; 8% of all prescriptions) (Figure 1). The types of macrolides also differed between countries – Estonian physicians preferred claritromycin (127 per 1000; Sweden 0.6 per 1000) but erythromycin was mainly prescribed in Sweden (21 per 1000, Estonia 3 per 1000).

Cephalosporins were the third most commonly used agents in Estonia (16%; 100 per 1000) but were less often prescribed in Sweden (7.5%; 26 per 1000) (Figure 1). First generation cephalosporins (cefalexin, cefadroxil) were prescribed with equal frequency in both countries whereas the second-generation cephalosporins (cefuroxime, cefprozil) were more frequently used in Estonia compared to Sweden (68 vs. 1.4 per 1000, respectively). No third- or fourth-generation cephalosporins were used in Estonia but they were prescribed occasionally in Sweden.

Among other antibacterials, nitrofurantoin, quinolones and lincosamides were rarely prescribed in either country. Sulfonamides and trimetoprim was more often prescribed in Estonia (8%; 50 per 1000) than in Sweden (4.7%; 17 per 1000). Aminoglycosides were not prescribed for Estonian children and very rarely used in Sweden. In Swedish adolescents, tetracyclines were used twice as often than in Estonian 12-17 year olds (30 vs. 16 per 1000; Figure 1).

### Selection of antibiotics by Estonian physicians and adherence to treatment guideline

GPs were responsible for the majority (73%), paediatricians for 12% and ear, nose and throat (ENT) physicians for 9% of prescriptions. Other specialists made the remaining 6% of all prescriptions.

The most common diagnoses for which antibiotics were prescribed were acute bronchitis (ICD-10 code J20; 20% of all antibiotic prescriptions), non-suppurative AOM (H65; 16%) and acute tonsillitis (J03; 12%). Other common diagnoses were acute laryngitis and tracheitis (J04; 8.3%), acute pharyngitis (J02; 7.8%), croup and epiglottitis (J05; 7.2%); acute sinusitis (J01; 5.9%) and diseases of the genitourinary system (N00-N99; 3.8%) There were 2% of prescriptions for skin and soft tissue infections (L.00-L.30) and 2.4% for pneumonia (J.13-J.18) (WHO International Classification of Diseases, http://www.who.int/classifications/icd/en/).

For acute bronchitis, 17 different antibiotics were prescribed in Estonia despite the guideline recommendation not to use antibiotics at all. Clarithromycin was the most commonly prescribed antibiotic, followed by amoxicillin and amoxicillin + clavulanic acid (Table 2).

**Table 2:** **Most common diagnoses in Estonia for which antibiotics are prescribed, guideline recommendations and the selection of drugs by physician speciality – percentage of prescriptions for specific diagnosis by speciality and No of prescriptions**

Diagnosis (Total No of prescriptions)	Primary recommendation in guidelines	Alternative recommendation in guidelines	GPs	Paediatricians	Ear, nose and throat physicians
**Acute bronchitis** (n = 31,670)	Antibiotics not indicated	NA	Clarithromycin (45%; n = 11,658)	Clarithromycin (50.5%; n = 2,342)	Clarithromycin (59%; n = 39)
			Amoxicillin (28%; n = 7,243)	Amoxicillin (22%; n = 1,040) Amoxicillin + clavulanic acid (9%; n = 418)	Amoxicillin (18%; n = 12) Amoxicillin + clavulanic acid (9% n = 6)
			Amoxicillin + clavulanic acid (10%; n = 2,486)		
**Non-suppurative otitis media (** n = 26,130)	Amoxicillin or penicillin	Macrolide if penicillin allergy	Amoxicillin (46%; n = 7,616)	Amoxicillin (49%; n = 1,432)	Amoxicillin (41%; n = 2,522)
			Amoxicillin + clavulanic acid (24%; n = 3,859)	Amoxicillin + clavulanic acid (17%; n = 497)	Amoxicillin + clavulanic acid (21%; n = 1306)
			Cefprozil (11%; n = 1,757)	Cefuroxim (13%; n = 381)	Cefprozil (12%; n = 742)
**Acute tonsillitis** (n = 18,890)	Phenoxymethylpenicillin or amoxicillin	Macrolide if penicillin allergy	Amoxicillin (30%; n = 4,443)	Phenoxymethylpenicillin (27%; n = 719)	Clarithromycin (24%; n = 228) Cefadroxil (16%; n = 145) Amoxicillin + clavulanic acid (14%; n = 131)
			Cefadroxil (18%; n = 2,598)	Amoxicillin (27%; n = 719) Amoxicillin + clavulanic acid (13%; n = 339)	
			Phenoxymethylpenicillin (15%; n = 2,201)		

For non-suppurative AOM, also 17 different antibiotics were prescribed, most commonly amoxicillin (in accordance with the guidelines) and amoxicillin + clavulanic acid, followed by cefprozil, cefuroxime and clarithromycin. There were 213 prescriptions per 1000 children less than 2 years of age and 87 prescriptions per 1000 for 2 to 18 years old children.

For acute tonsillitis, amoxicillin was most commonly prescribed, followed by phenoxymethylpenicillin; both were recommended by guidelines as first line treatment (Table 2).

The antibiotic preference for acute bronchitis was the same between GPs, paediatricians and ENT specialists. Similarly, for non-purulent AOM, the choices coincided, except that GPs and ENT physicians preferably prescribed the second-generation cephalosporin cefprozil whereas paediatricians preferred cefuroxime. For acute tonsillitis, GPs predominantly prescribed amoxicillin, paediatricians prescribed phenoxymetylpenicillin and amoxicillin equally often whereas ENT physicians predominantly prescribed clarithromycin (Table 2).

The antibiotics prescriptions for Estonian children were equally divided between male and female subjects. Slightly more prescriptions were purchased for boys less than 2 years old compared to the girls at the same age and for adolescent girls compared to the adolescent boys (55% vs 45% for both age groups).

The frequency of prescriptions for systemic antibiotics per unique child purchased in 2007 in Estonia is described in the Table 3.

**Table 3:** **The frequency of prescriptions for systemic antibiotics per unique child prescribed in 2007**

Number of prescriptions for systemic antibiotics for child per year	Frequency (Number of children with prescription)	Percentage of children with prescription	Cumulative percentage
1 prescription	51849	57.67	57.67
2 prescriptions	21235	23.62	81.29
3 prescriptions	9105	10.13	91.42
4 prescriptions	4128	4.59	96.01
5 prescriptions	1898	2.11	98.12
6 prescriptions	892	0.99	99.11
7 prescriptions	413	0.46	99.57
8 or more prescriptions	387	0.43	100

## Discussion

While comparing antibiotic consumption in ambulatory setting in Estonia and Sweden we observed that in both countries the paediatric antibiotic use is in low ranges (616 in Estonia and 353 in Sweden) as compared to Spain or France (over 1500 and 1000 prescriptions per 1000 children, respectively) (Sharland 2007). However, the Estonian children received twice as many prescriptions for antibacterials compared to Swedish counterparts. Secondly, similarly to a study conducted almost two decades ago in Estonian and Swedish University Hospitals (Kiivet et al. 1998) we noted differences in antibiotic prescription preferences in the two countries – most conspicuously, phenoxymethylpenicillin covered half of the prescriptions in Sweden, whereas amoxicillin and clarithromycin were preferred in Estonia.

The quantitative differences in antibiotic use between countries could not be explained by dissimilarities in resistance level of common outpatient microorganisms, which have been reported to be low in general in both countries. For example, in 2005 to 2009, the proportion of methicillin resistant *S. aureus* has ranged between 2% to 9% in Estonia and 0.5% to 1% in Sweden; no penicillin resistant *S. pneumoniae* among invasive strains has been reported in Estonia and the rate in Sweden was 0.1% to 2.5% (European Centre for Disease Prevention and Control 2999). A slightly higher number was reported among colonising strains of *S. pneumoniae* in Estonia; 6% of all isolates of *S. pneumoniae* were either penicillin-nonsusceptible *Streptococcus pneumoniae* (PNSP) or resistant to erythromycin, but none showed resistance to penicillin (Tamm et al. 2007). Macrolide resistance of *S. pyogenes* is about 5% in Estonia (European Centre for Disease Prevention and Control 2999).

Our study brought also out the qualitative differences of ambulatory antibiotic use in children between two countries suggesting that in countries with low rates of antibiotic resistance among outpatient pathogens (e.g. Estonia) narrow spectrum penicillins could be used instead of wide spectrum agents. The reasons why Estonian physicians tend to prescribe wide spectrum agents have not been systematically studied but the likely reasons could involve the relatively liberal guideline recommendations. For example, for acute tonsillitis, the Estonian guidelines, in addition to phenoxymethylpenicillin recommend amoxicillin despite the fact that *S. pyogenes* is uniformly susceptible to penicillin (Hraoui et al. 2011). Other reasons could involve the pressure from parents to receive the newest agents, a limited option for etiologic diagnosis in outpatient setting, the promotional activities of pharmaceutical industry, the lack of detailed knowledge due to poor dissemination of guidelines or simply poor adherence to guidelines (Hedin et al. 2006). The latter reason could apparently be supported by the significant amount (20%) of antibiotic prescriptions for acute bronchitis, a disease not requiring antibiotic treatment at all. Poor adherence to guidelines is also suggested by the wide use of macrolides in Estonia although they are only recommended for patients with penicillin allergy or high use of oral cephalosporins, which are the second or third choice agents according to the Estonian guideline. We also show that in Estonia 65% of prescriptions for trimetoprim-sulfamethoxazol were for acute upper respiratory infections despite the indications being exacerbation of chronic bronchitis and genitourinary infections.

(Lusini et al. Secondly, macrolides were extensively used in Estonia vs. Sweden, accounting for 24% and 8% of all prescriptions, respectively. The extended use of macrolides has been related to increased carriage of penicillin non-susceptible *S.pneumoniae*^2009^) and the inappropriate prescription of macrolides was one of the targets for the STRAMA programme (Mölstad et al. 2008). The choice of macrolide also differed between countries, clarithromycin predominating in Estonia while the parent drug erythromycin was mainly used in Sweden. The preference of clarithromycin by Estonian prescribers could possibly be explained by the easier administration scheme - twice as compared to four times daily. Slightly better tolerability in terms of gastrointestinal side effects of clarithromycin compared to erythromycin has also been reported (Lee et al. 2008).

Similar to other studies (Otters et al. 2004; Finkelstein et al. 2000) almost a half of prescriptions were made for respiratory tract infections like bronchitis, laryngitis, pharyngitis, AOM and sinusitis which rarely are caused by bacteria - and for which Estonian guidelines recommend not to use antibiotics or - for AOM - suggest to use ‘wait and see strategy’ in children above 2. The reasons for this non-adherence to guidelines are probably similar to those suggested above but they have not been systematically studied.

### Study strengths and limitations

The strength of this study is the lack of selection bias as the databases cover the entire paediatric populations in Estonia and Sweden. Although the data collected in Estonia are more detailed than that collected in Sweden, the method and reliability of data collection is similar between the countries, offering the potential for comparative estimates of the extent of antibiotic use by age groups.

An apparent weakness of the study comes from the fact that the Swedish data collection is not diagnose-linked so we were not able to assess the guideline adherence in Sweden.

Nor was it possible to describe between-country variability of dose regimens and duration of treatment. In order to study between-country variability in treatment practices including choice of antibiotics for different conditions, a prospective study would be required.

One possible limitation could be the fact that we used only data from one year. As our main aim was to compare the prescription preferences of paediatric outpatient antibiotic use, which is more stable over years than the total consumption (Kiivet et al. 1998; Sharland 2007) we assume that this will not affect the reliability of our study.

## Conclusion

The low overall consumption of antibiotics is no indicator of appropriate paediatric antibiotic prescribing. The higher rate of antibiotic use in Estonia and the apparent high use of extended spectrum antibiotics emphasizes the need for national activities similar to the Swedish STRAMA programme in order to prevent further misuse, including the implementation of clinical pharmacy services. Guidelines for the treatment of infectious diseases should be regularly updated and actively distributed among all prescribing physicians. Also, auditing activities should focus on rational use of antibiotics and compliance to evidence based guidelines.

## Authors’ original submitted files for images

Below are the links to the authors’ original submitted files for images. Authors’ original file for figure 1 Authors’ original file for figure 2
