# Supplementary figures and images for: Antibiotic prescription preferences in paediatric outpatient setting in Estonia and Sweden
Source: Springerplus. 2013 Mar 21;2(1):124. doi: 10.1186/2193-1801-2-124 (PMC3647088; doi:10.1186/2193-1801-2-124)

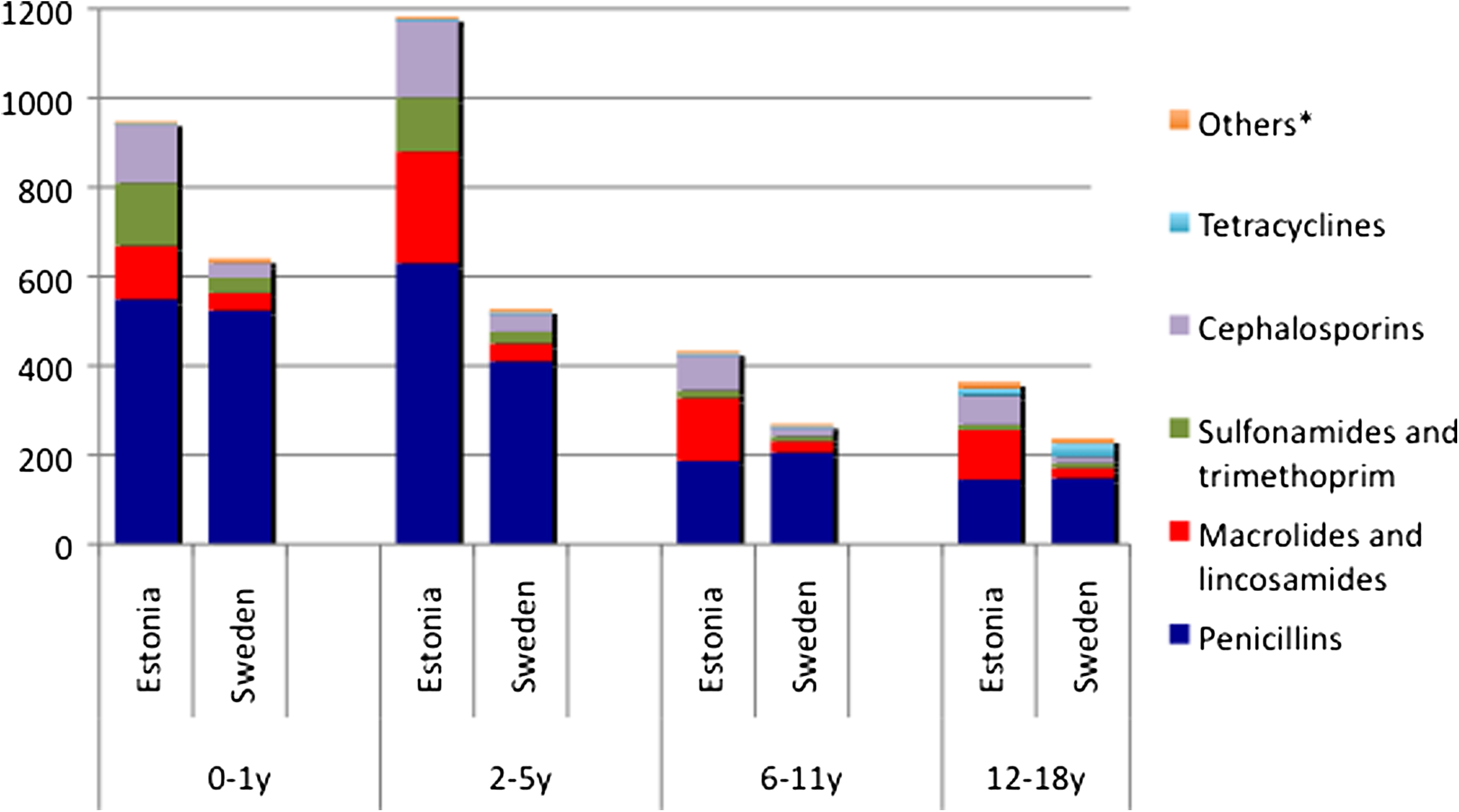

Supplement: Supplementary file 1 — Authors’ original file for figure 1 [file 40064_2012_224_MOESM1_ESM.tiff]
