# Supplementary material for: Antibiotic prescription preferences in paediatric outpatient setting in Estonia and Sweden
Source: Springerplus. 2013 Mar 21;2(1):124. doi: 10.1186/2193-1801-2-124 (PMC3647088; doi:10.1186/2193-1801-2-124)
Supplement: Supplementary file 2 — Authors’ original file for figure 2 [file 40064_2012_224_MOESM2_ESM.pdf]

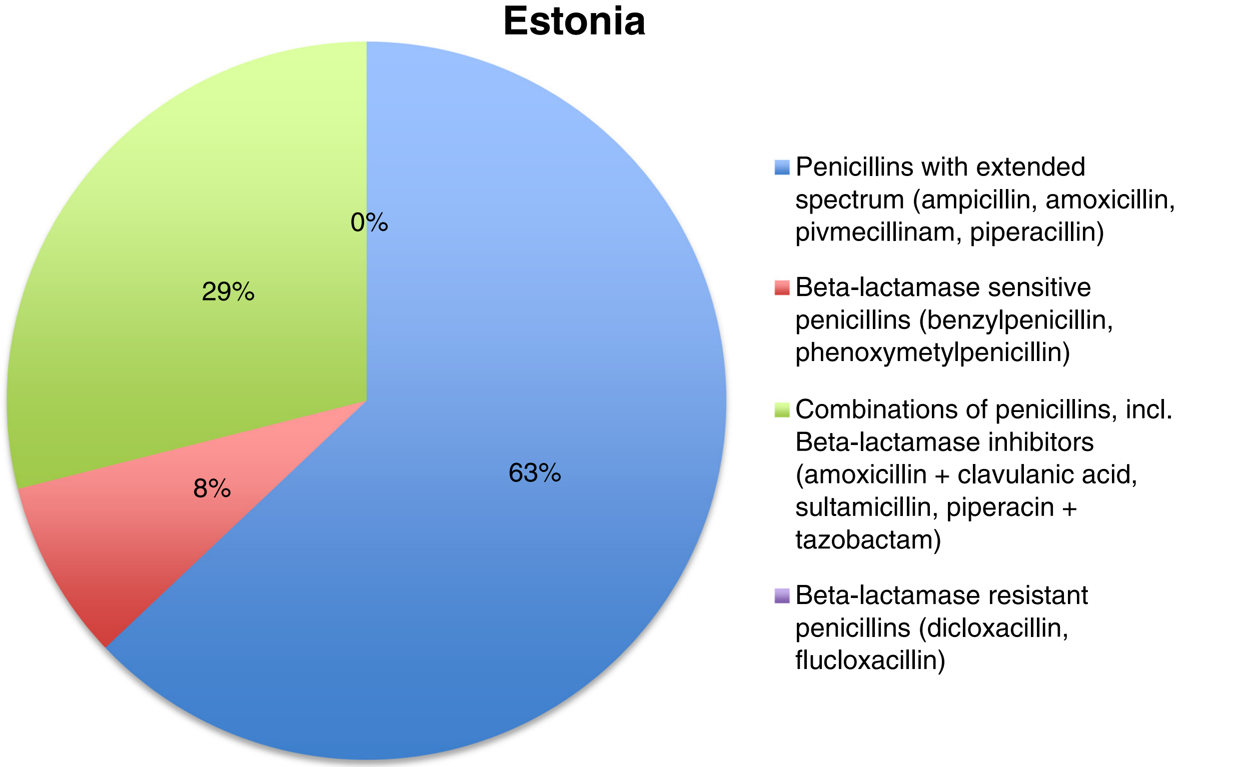
Estonia
0%
29%
8%
63%
Penicillins with extended spectrum (ampicillin, amoxicillin, pivmecillinam, piperacillin)
Beta-lactamase sensitive penicillins (benzylpenicillin, phenoxymetylpenicillin)
Combinations of penicillins, incl. Beta-lactamase inhibitors (amoxicillin + clavulanic acid, sultamicillin, piperacin + tazobactam)
Beta-lactamase resistant penicillins (dicloxacillin, flucloxacillin)

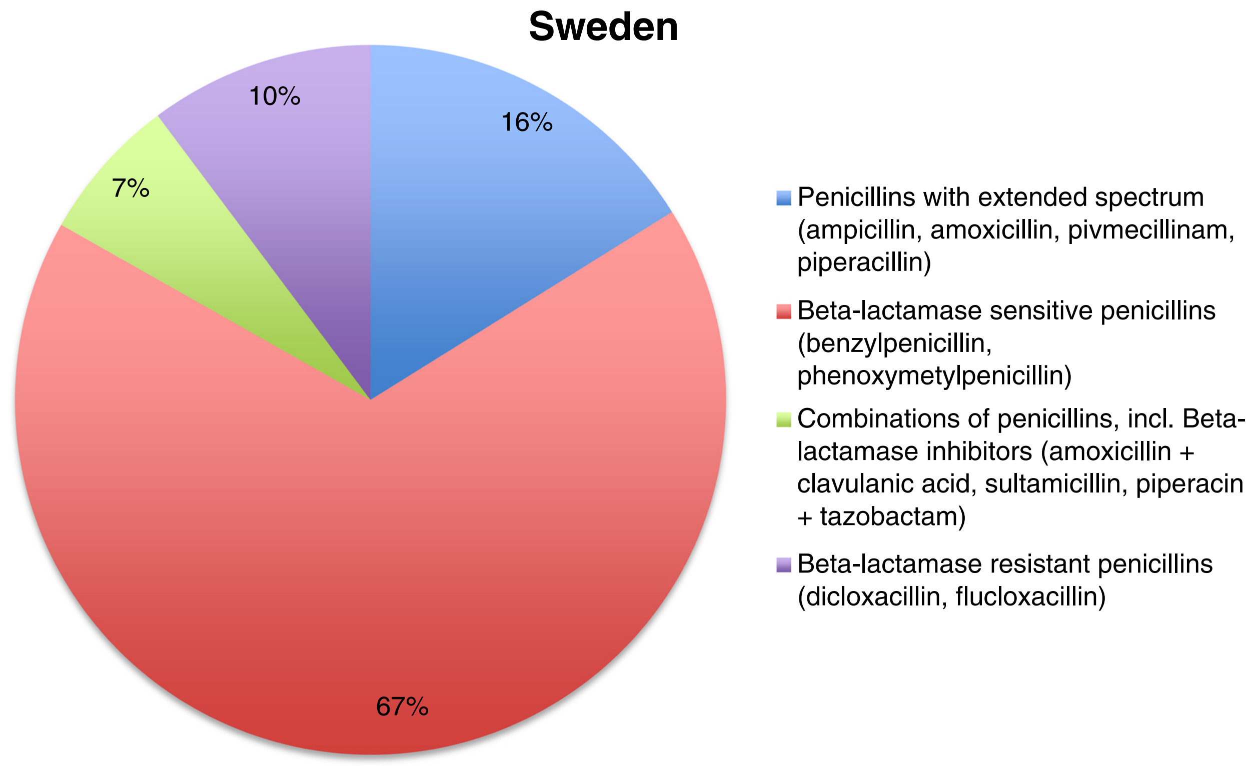
Sweden
10%
16%
7%
67%
Penicillins with extended spectrum (ampicillin, amoxicillin, pivmecillinam, piperacillin)
Beta-lactamase sensitive penicillins (benzylpenicillin, phenoxymetylpenicillin)
Combinations of penicillins, incl. Beta-lactamase inhibitors (amoxicillin + clavulanic acid, sultamicillin, piperacin + tazobactam)
Beta-lactamase resistant penicillins (dicloxacillin, flucloxacillin)
